# Protective and Risk Factors for Medical and Nursing Staff Suffering From Psychological Symptoms During COVID-19

**DOI:** 10.3389/fpsyg.2021.603553

**Published:** 2021-04-16

**Authors:** Hailong Luo, Huiqi Yao, Yuandi Xi, Zhun Zhang, Jia Li, Jie Li, Xuewen Wang, Zhixiong Zhong, Yan Lv

**Affiliations:** ^1^Department of Psychology, Meizhou People's Hospital, Meizhou, China; ^2^Department of Rehabilitation, Meizhou People's Hospital, Meizhou, China; ^3^Department of Epidemiology, Public Health College, Capital Medical University, Beijing, China; ^4^Department of Psychology, Hainan General Hospital, Haikou, China; ^5^Department of Geriatrics, Meizhou People's Hospital, Meizhou, China; ^6^Department of Cardiology, Wuhan Asian Cardiovascular Hospital, Wuhan, China; ^7^Medical Department, Meizhou People's Hospital, Meizhou, China; ^8^Department of Neurology, Hainan General Hospital, Haikou, China

**Keywords:** medical staff, social support, COVID-19, psychological symptoms, protective elements

## Abstract

**Background:** With the outbreak of the coronavirus disease 2019 (COVID-19) epidemic in China, the general public but also medical staff were confronted with psychological challenges, suffering from the highly infectious and unknown characteristics of COVID-19. In this study, we surveyed psychological symptoms including anxiety, depression, and sleep disorders in medical staff.

**Method:** A questionnaire star/WeChat link-based survey assessing the Generalized Anxiety Disorder 7-item scale, Patient Health Questionnaire-9 depression, the Insomnia Severity Index, Social Support scales in addition to lifestyle, and income level was conducted and included 8,288 medical staff from 24 provinces in China. Pearson Chi-square and Mann-Whitney *U*-tests were used to evaluate single risk factors and significant differences in psychological symptoms before and during the outbreak of COVID-19. Binary logistic regression analyses were conducted for the risk factors of anxiety, depression, and sleep disorder symptoms.

**Results:** Medical staff had a high incidence of psychological symptoms, which was more prominent during the COVID-19 epidemic. Comparatively, females, nurses, first-line department, never exercised, and low income were risk factors for psychological symptoms. Social support including objective support, subjective support, support utility, and regular sports over 3 times per week were protective and manageable elements that could protect from and manage the psychological symptoms of medical staff.

**Conclusion:** The susceptibility of psychological symptoms among medical staff should be of concern to policymakers and the public in the long-term, and the aggravation of mental health problems of medical staff could be eased by providing adequate social support during and after the COVID-19 outbreak.

## Background

At the beginning of 2020, coronavirus disease 2019 (COVID-19) broke out in Wuhan, China; 81,062 patients were diagnosed and 3,204 patients died according to one report (Deng and Peng, 2020). Although China had previously experienced the severe acute respiratory syndrome epidemic (Qiu et al., 2018), the Chinese people were still caught off guard with COVID-19 (Huang C. et al., 2020). The Chinese government learned from previous epidemics (Zhang, 2020) and developed effective strategies for controlling COVID-19 by isolating cases and contact tracing (Hellewell et al., 2020). Prior to the onset of other inflection points, Doctor Wengliang Li strongly encouraged Chinese medical staff in the battle against COVID-19. However, COVID-19 still posed a challenge in Chinese society and medical staff standing on the frontline treating patients were suffering from pressure and phobia (Asmundson and Taylor, 2020).

COVID-19 was not only an infectious attack (Jin et al., 2020; Luo et al., 2020), but also resulted in a considerable mental health burden in the general Chinese population (Du, 2020) and among health care workers at the beginning and peak of the pandemic. Anxiety, depression, and sleep disorders were the most common psychological symptoms in frontline medical staff under stress (Yaribeygi et al., 2017) including emergency departments (Song et al., 2020), ICU (Hu et al., 2021), and territory hospitals (Huang J. Z. et al., 2020; Fu et al., 2021). Medical staff, including students and caregivers (Paiva et al., 2018), and professionals (Zerbini et al., 2020) were potentially at risk of developing psychological symptoms, which would affect their daily life and work. Notably, the medical and nursing staff working in Wuhan had a high incidence of mental health disturbances in the immediate wake of the viral epidemic (Kang et al., 2020). A similar situation was found in European countries (Hummel and Oetjen, 2021).

Medical staff were the backbone of the battle against the COVID-19 epidemic. For this battle to be successful, the psychological well-being of medical personnel was essential (Kang et al., 2020) and will have an impact even after the pandemic (Juan et al., 2020; Moreno et al., 2020). Researchers worked diligently to determine risk factors and protective elements for medical staff confronting psychological symptoms. Among many factors such as marriage status, gender, and age, social support was one of the intervenable elements. At the same time, coping strategies were suggested based on those studies (Chen et al., 2021). Social support could improve the psychological health of caregivers such as nurses (Pedro et al., 2008). Therefore, we conducted a questionnaire star/WeChat link-based survey of the common psychological symptoms of medical staff as well as demographic factors including sex, profession, lifestyle, and social support to obtain an adoptable method to improve the psychological health conditions in the epidemic background not only for Chinese medical staff but also health workers around the world.

## Methods

### Subjects and Data Collection

An electronic WeChat-based survey was distributed to medical staff from 24 provinces in China. The questionnaire survey was carried out from February 18, 2020 to May 7, 2020. The 24 provinces in this survey included: Guangdong, Hubei (except Wuhan), Wuhan, Hainan, Jiangxi, Beijing, Henan, Hebei, Shanxi, Hunan, Jiangsu, Zhejiang, Xinjiang, Anhui, Sichuan, Fujian, Guangxi, Shanghai, Tianjin, Liaoning, Shandong, Heilongjiang, Shanxi, and Hong Kong. There were 1,911,317 doctors and 3,020,813 nurses in China according to the 2018 annual survey (see Supplementary Material), the province-specific data of the number of medical staff were not statistically available since the government simply divided those areas into several districts, and the data had not been taken into consideration. The sample size in our survey was 8,028, which accounted for 0.16% of the total of doctors and nurses in China. The questionnaire consisted of 36 items; the average time for the test was about 200 s and responses <60 s were excluded. The participants were largely classified into doctors and nurses, those categorized as belonging to medical staff but classified into other afflicted professions were excluded. Questionnaires reporting paradoxical answers for the same question (questions 4 and 36) were also excluded.

### Ethical Aspects

The Research Ethics Committee of Hainan General Hospital approved the study protocol. All participants indicated their agreement to participate in the study via the electronic informed consent included in the survey.

### Calculation of Sample Size

Estimates suggest that ~60% of the population of physicians who work in hospitals presented with psychological symptoms (Shanafelt et al., 2003). Accepting an estimate of absolute precision (i.e., how close the estimate is to the true value) of 10% and a level of significance of 1%, the minimum estimated sample size was 160 physicians 8. Our sample was larger than this and the survey not only sought to explore the prevalence of anxiety, depression, and sleep disorders, but also provided suggestions to the participants along with an evaluation of how to better manage psychological symptoms.

### Questionnaire and Evaluation

The questionnaire asked for participants' gender, profession (doctor or nurse), marital status, income level, lifestyle including smoking, alcohol, frequency of exercise, and other things significant to a medical history. The Social Support Scale (SSS) was also used to examine the underlying causes and solutions of psychological symptoms. The SSS (Supplementary File, Data Sheet 1) was developed by Professor Xiao Shuiyuan, which included objective support, subjective support, and support utility (Yuan, 1994). Medical staff were also classified as belonging to first-line departments like the intensive care unit (ICU), fever clinic, emergency, infectious diseases, respiratory unit, and critical care, and second-line departments which included other clinical departments. The Generalized Anxiety Disorder 7-item (GAD-7), Patient Health Questionnaire-9 (PHQ-9) depression, and Insomnia Severity Index (ISI) scales were used for the evaluation of psychological symptoms. GAD-7 scores ≥5 were considered positive in measuring anxiety; PHQ-9 scores ≥5 were considered positive in measuring depression; ISI scores ≥8 were considered positive for measuring sleep disorders. After the survey, participants were provided with a survey evaluation and given suggestions to reduce anxiety, depression, and sleep disorders to help them cope with their problems. The Chinese and English versions of the questionnaires are included (Data Sheet 2, 3). We designed the questionnaire to cover both pre- and post-epidemic periods to compare the psychological symptoms of medical staff. So, the population was the same.

### Statistical Analyses and Results Presentation

Variables were individually compared based on diagnoses of anxiety (yes/no), depression (yes/no), and sleep disorders (yes/no). Categorical and continuous variables were analyzed using the Pearson Chi-square test and Mann-Whitney *U*-test, respectively. Pairwise comparisons of statistically significant data from the Pearson Chi-square test were performed using the *Z*-test and the Bonferroni method was used to obtain the *p*-value. The Sigma Plot software was used to compare social support between groups. Variables associated with *p* < 0.10 in the univariate analyses were included in a binary logistic regression model to identify the risk factors of anxiety, depression, and sleep disorders. A two-tailed *p*-value of < 0.05 was considered statistically significant. All statistical analyses were conducted using SPSS for Windows version 23 (SPSS, Inc., Chicago, IL, USA). Cytoscape 3.6 was used for the presentation of the statistical results (Shannon et al., 2003).

## Results

### Population Description

**Figure 1:**
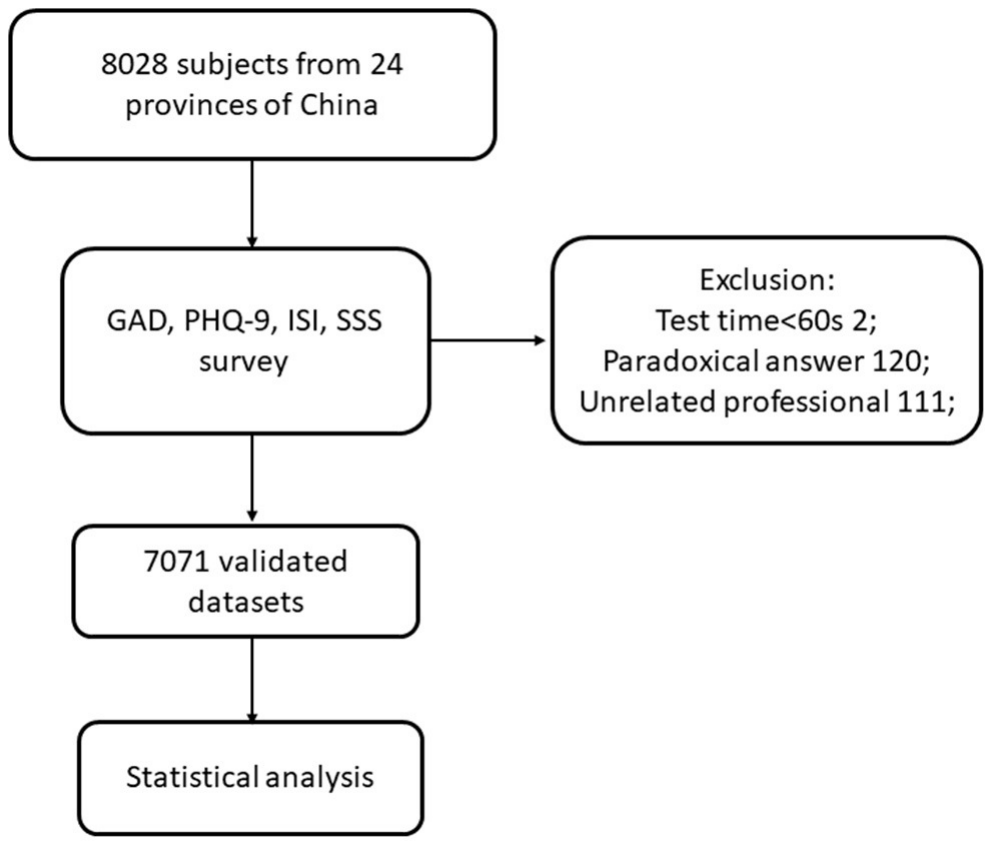
Cohort diagram.

There were 8,082 medical workers from 24 provinces of China who participated in the survey. Of these, 7,071 questionnaires from doctors and nurses were included for further statistical analysis. The Consolidated Standards of Reporting Trials flow diagram is shown in Figure 1. There were 2,037 males (29%), and 5,034 females (71%). A large proportion of participants were in the 21–30 years age group, accounting for 43% of all participants. There were 3,693 doctors (52%) and 3,378 nurses (48%), 5,069 (72%) were married, and 2,549 (36%) were doctors and nurses from first-line departments. Most doctors and nurses (*n* = 3,288, 46%) were paid RMB 50,000–100,000 annually. Participants' demographic characteristics are shown in Table 1.

**Table 1:** Demographic characteristics of the participants.

**Variables**	**Participants**	**Percentage**
**Gender**		
Male	2,037	29%
Female	5,034	71%
**Age (years)**		
≤ 20	126	2%
21–30	3,021	43%
31–40	1,908	27%
41–50	1,711	24%
51–60	284	4%
>60	21	0%
**Profession**		
Doctor	3,693	52%
Nurse	3,378	48%
**Department**		
First line	2,549	36%
Second line	4,522	64%
**Income (renminbi/per year)**		
<50,000	3,127	44%
50,000–100,000	3,288	46%
110,000–150,000	460	7%
160,000–200,000	113	2%
210,000–300,000	55	1%
310,000–400,000	16	0%
>400,000	12	0%
**Regular sports activities**		
Never exercise	963	14%
Irregular physical activity	4,080	58%
Get more than 20 min of exercise twice a week	823	12%
Get more than 20 min of exercise 3–4 times a week	680	10%
Get more than 20 min of exercise over 5 times a week	525	7%
**Smoking**		
Smoking	606	9%
No-smoking	6,465	91%
**Alcohol consumption**		
Alcohol	311	4%
No-alcohol	6,760	96%
**Marital status**		
Unmarried	1,852	26%
Married	5,069	72%
Divorced	134	2%
Death of a spouse	11	0%
Cohabitation	5	0%

### Anxiety, Depression, and Sleep Disorder Scores and Prevalence Before and During COVID-19

**Figure 2:**
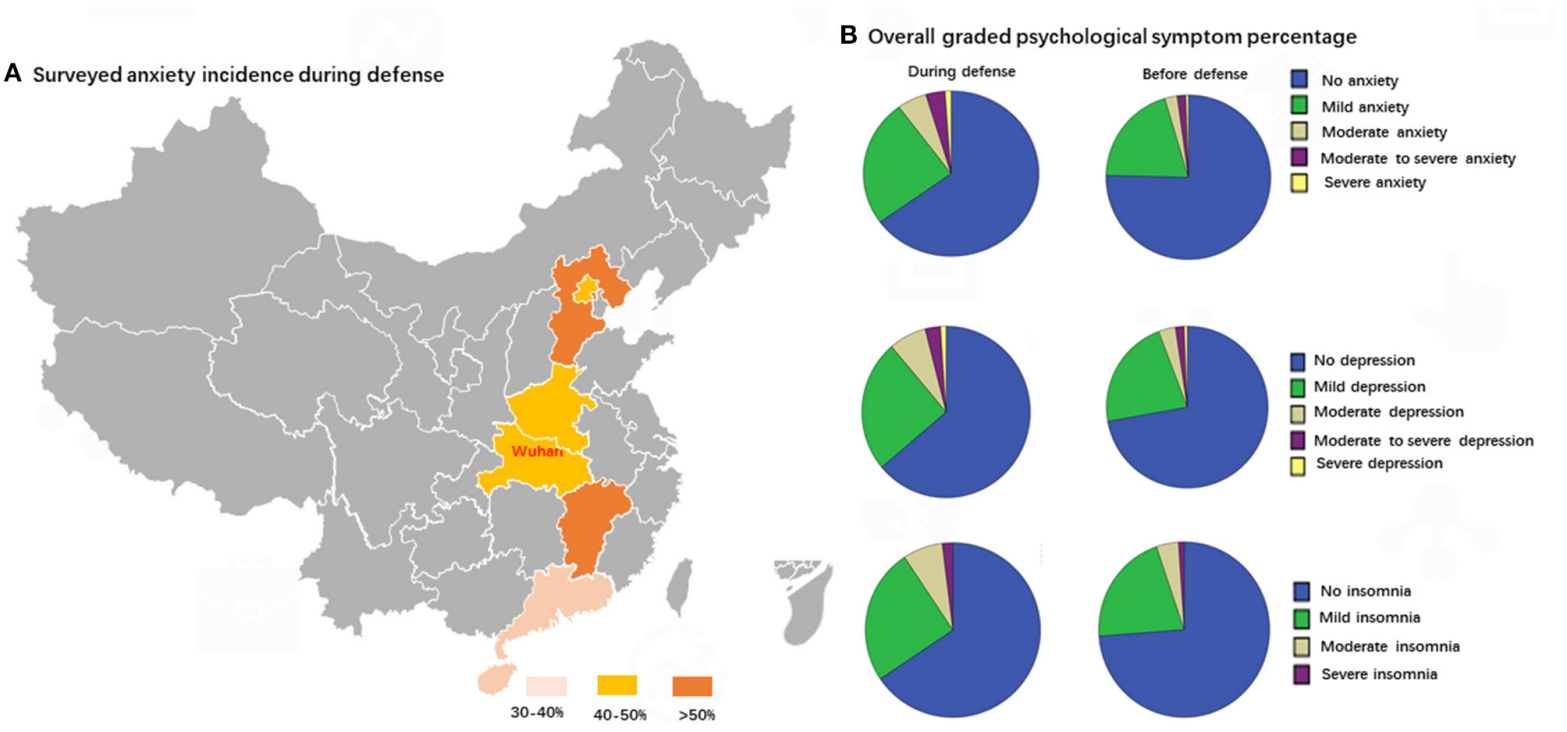
Psychological symptom percentage and anxiety incidence distribution.

Due to the imbalance of the samples in the provinces, we did not carry out statistical comparisons of the incidence of psychological symptoms of the surveyed medical staff among provinces (Figure 2A). The overall incidence of anxiety among the Chinese medical staff was 34.7% and the mild anxiety incidence was 24.8%. The district distribution of anxiety incidences is presented in Figure 2A: the incidence for Wuhan was 40%, and the incidences for Guangdong and Hainan were under 40%, several provinces had an incidence over 50%. However, the sample of surveyed participants in most provinces was small and should be under-estimated. Compared to the previous period, the percentages of anxiety, depression, and sleep disorders were evidently higher during the COVID-19 outbreak when compared with before. The most significant increase in anxiety, depression, and sleep disorders was clustered in the mild grade as illustrated in Figure 2B.

**Figure 3:**
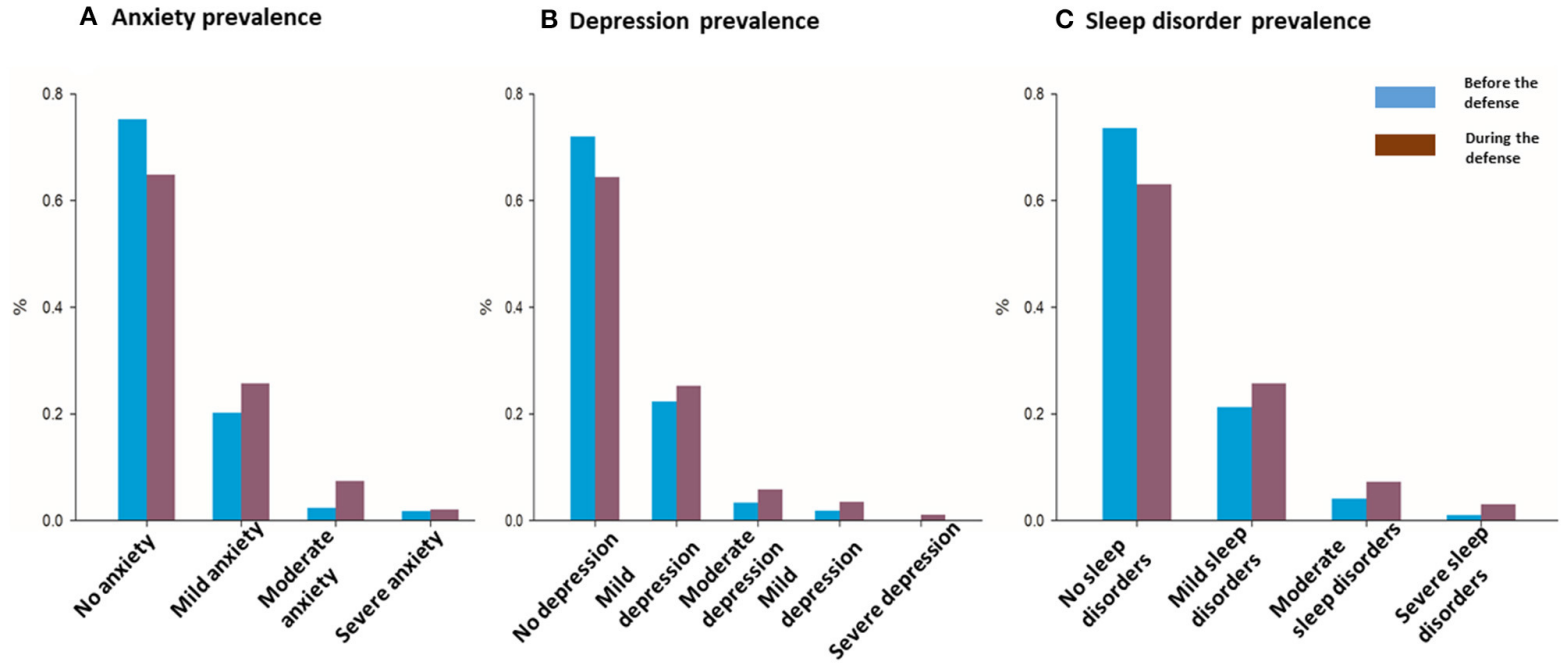
Anxiety, depression, and sleep disorders incidence before and during COVID-19 outbreak.

As indicated in Figure 3, the incidence among the Chinese medical staff of anxiety, depression, and sleep disorders were 35, 36, and 37%, respectively, during COVID-19, and 25, 28, and 26%, respectively, before the outbreak. The anxiety, depression, and sleep disorders percentages were evidently higher during the pandemic compared with before. The graded anxiety, depression, and sleep disorders percentages were similar to the whole psychological symptom spectrum.

### Multivariate Analyses

**Figure 4:**
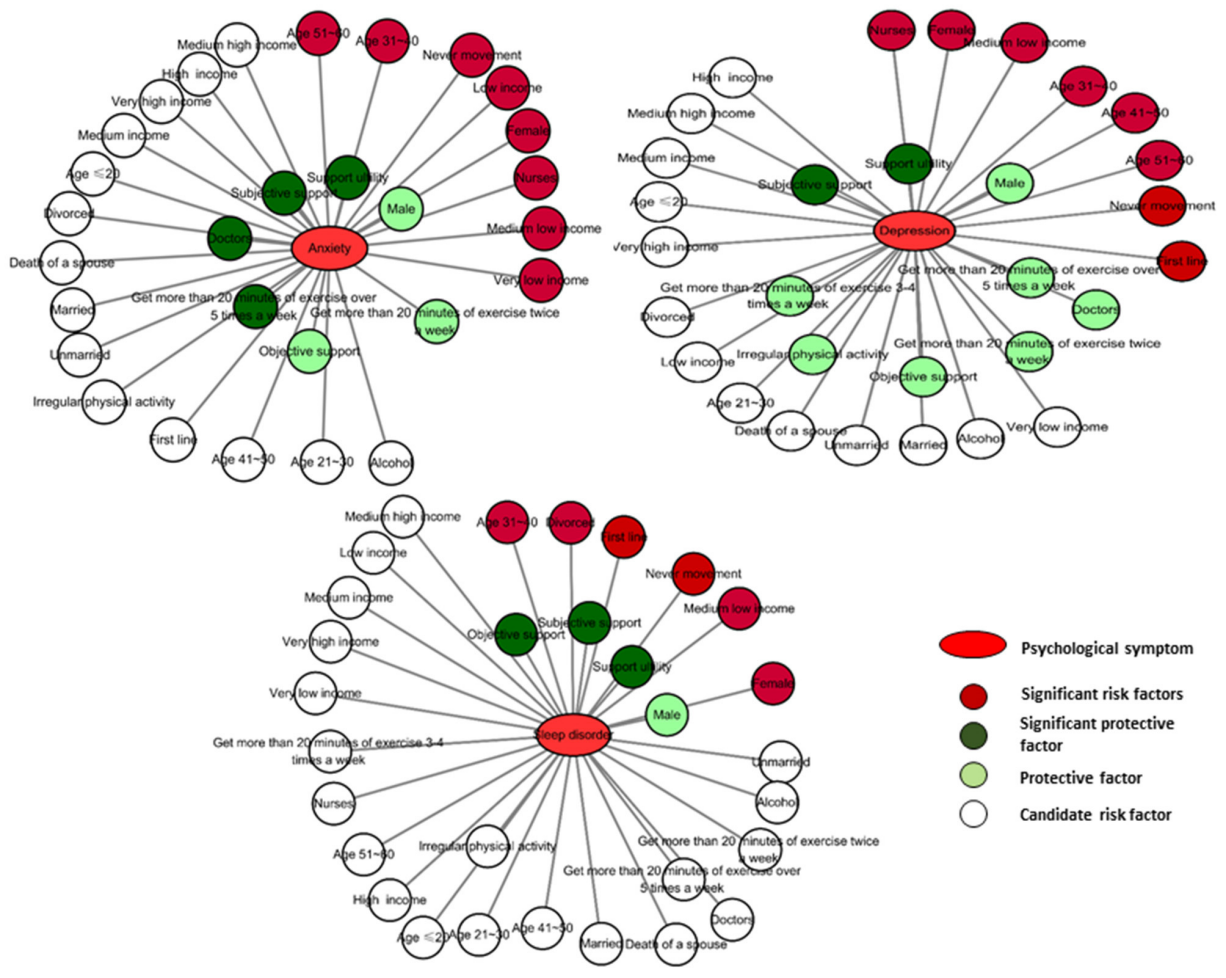
Roadmap of risk factors and protective elements for psychological symptoms of medical staff during COVID-19 pandemic.

All variables with *p* < 0.10 in the univariate analyses (Table 2) were included in binary logistic regression models for each analyzed outcome. Those working in first-line departments such as the ICU, fever clinic, emergency, infectious diseases, respiratory, and critical care were more likely to develop anxiety (OR = 1.979, *p* < 0.01), depression (OR = 1.468, *p* < 0.01), and sleep disorders (OR = 1.979, *p* < 0.01). Medical staff who never exercised were also susceptible to anxiety (OR = 2.045, *p* < 0.01), depression (OR = 1.979, *p* < 0.01), and sleep disorders (OR = 1.557, *p* < 0.01). The three dimensions of social support of objective support, subjective support, and support utility could protect the medical staff from suffering from anxiety, depression, and sleep disorders, as shown in Tables 2, 3. Gender, marital status, profession, income level, smoking, and alcohol presented group differences but failed to achieve significant correlations with the psychological symptom outcomes. The candidate risk factors were grouped into candidate, protective, significant protective, and significant risk factors for anxiety, depression, and sleep disorders based on the statistical results (Figure 4).

**Table 2:** Variables of psychological symptoms of Chinese medical staff during COVID-19.

**Variable**	**Anxiety**				**Depression**				**Sleep disorders**			
	**No *N* (%)/x(sd)**	**Yes *N*(%)/x(sd)**	***X*^**2**^/*U***	***p***	**No *N*(%)/x(sd)**	**Yes *N*(%)/x(sd)**	***X*^**2**^/*U***	***p***	**No *N*(%)/x(sd)**	**Yes *N*(%)/x(sd)**	***X*^**2**^/*U***	***p***
**Gender**			9.656	0.002			5.481	0.019			2.279	0.131
Male	1368(19.3)	669(9.5)			1327(18.8)	710(10.0)			1348(19.1)	689(9.7)		
Female	3184(45.0)	1850(26.2)			3130(44.3)	1904(26.9)			3236(45.8)	1798(25.4)		
Age (years)			12.765	0.026			69.373	0.000			9.516	0.090
≤ 20	80(1.1)^a^	46(0.7)^a^			71(1.0)^a^	55(0.8)^a^			79(1.1)^a^	47(0.7)^a^		
21–30	1967(27.8)^a^	1054(14.9)^a^			1822(25.8)^a^	1199(17.0)^b^			1975(27.9)^a^	1046(14.8)^a^		
31–40	1184(16.7)^a^	724(10.2)^b^			1150(16.3)^a^	758(10.7)^b^			1197(16.9)^a^	711(10.1)^b^		
41–50	1102(15.6)^a^	609(8.6)^a^			1182(16.7)^a^	529(7.5)^b^			1119(15.8)^a^	592(8.4)^a^		
51–60	203(2.9)^a^	81(1.1)^b^			213(3.0)^a^	71(1.0)^b^			197(2.8)^a^	87(1.2)^a^		
>60	16(0.2)^a^	5(0.1)^a^			19(0.3)^a^	2(0.0)^b^			17(0.2)^a^	4(0.1)^a^		
**Profession**			14.505	0.000			5.657	0.017			10.469	0.001
Doctor	2454(34.7)^a^	1239(17.5)^b^			2376(33.6)^a^	1317(18.6)^b^			2459(34.8)^a^	1234(17.5)^b^		
Nurse	2098(29.7)^a^	1280(18.1)^b^			2081(29.4)^a^	1297(18.3)^b^			2125(30.1)^a^	1253(17.7)^b^		
**Departmen**t			24.108	0.000			38.346	0.000			56.154	0.000
First-line	1546(21.9)^a^	1003(14.2)^b^			1486(21.0)^a^	1063(15.0)^b^			1508(21.3)^a^	1041(14.7)^b^		
Second-line	3006(42.5)^a^	1516(21.4)^b^			2971(42.0)^a^	1551(21.9)^b^			3076(43.5)^a^	1446(20.4)^b^		
**Income (per year)**			33.686	0.000			16.782	0.010			16.632	0.011
<50,000	2081(29.4)^a^	1046(14.8)^b^			1988(28.1)^a^	1139(16.1)^a^			2052(29.0)^a^	1075(15.2)^a^		
50,000–100,000	2101(29.7)^a^	1187(16.8)^a^			2102(29.7)^a^	1186(16.8)^a^			2151(30.4)^a^	1137(16.1)^a^		
110,000–150,000	247(3.5)^a^	213(3.0)^b^			253(3.6)^a^	207(2.9)^b^			261(3.7)^a^	199(2.8)^b^		
160,000–200,000	65(0.9)^a^	48(0.7)^a^			67(0.9)^a^	46(0.7)^a^			67(0.9)^a^	46(0.7)^a^		
210,000–300,000	40(0.6)^a^	15(0.2)^a^			32(0.5)^a^	23(0.3)^a^			36(0.5)^a^	19(0.3)^a^		
310,000–400,000	10(0.1)^a^	6(0.1)^a^			8(0.1)^a^	8(0.1)^a^			9(0.1)^a^	7(0.1)^a^		
>400,000	8(0.1)^a^	4(0.1)^a^			7(0.1)^a^	5(0.1)^a^			8(0.1)^a^	4(0.1)^a^		
**Regular sports activities**			123.199	0.000			203.062	0.000			103.871	0.000
Never exercise	501(7.1)^a^	462(6.5)^b^			454(6.4)^a^	509(7.2)^b^			513(7.3)^a^	450(6.4)^b^		
Irregular physical activity	2591(36.6)^a^	1489(21.1)^a^			2521(35.7)^a^	1559(22.0)^b^			2617(37.0)^a^	1463(20.7)^a^		
Get more than 20 min of exercise twice a week	578(8.2)^a^	245(3.5)^b^			576(8.1)^a^	247(3.5)^b^			574(8.1)^a^	249(3.5)^b^		
Get more than 20 min of exercise 3–4 times a week	480(6.8)^a^	200(2.8)^b^			498(7.0)^a^	182(2.6)^b^			484(6.8)^a^	196(2.8)^b^		
Get more than 20 min of exercise over 5 times a week	402(5.7)^a^	123(1.7)^b^			408(5.8)^a^	117(1.7)^b^			396(5.6)^a^	129(1.8)^b^		
**Smoking**			1.517	0.218			0.942	0.332			0.010	0.919
Smoking	404(5.7)	202(2.9)			393(5.6)	213(3.0)			394(5.6)	212(3.0)		
No-smoking	4148(58.7)	2317(32.8)			4064(57.5)	2401(34.0)			4190(59.3)	2275(32.2)		
Alcohol consumption			0.398	0.528			2.451	0.117			2.735	0.098
Alcohol	195(2.8)	116(1.6)			183(2.6)	128(1.8)			188(2.7)	123(1.7)		
No-alcohol	4357(61.6)	2403(34.0)			4274(60.4)	2486(35.2)			4396(62.2)	2364(33.4)		
**Marital status**			4.489	0.344			23.048	0.000			8.062	0.089
Unmarried	1225(17.3)^a^	627(8.9)^a^			1084(15.3)^a^	768(10.9)^b^			1212(17.1)^a^	640(9.1)^a^		
Married	3230(45.7)^a^	1839(26.0)^a^			3282(46.4)^a^	1787(25.3)^b^			3289(46.5)^a^	1780(25.2)^a^		
Divorced	87(1.2)^a^	47(0.7)^a^			82(1.2)^a^	52(0.7)^a^			72(1.0)^a^	62(0.9)^b^		
Death of a spouse	6(0.1)^a^	5(0.1)^a^			6(0.1)^a^	5(0.1)^a^			7(0.1)^a^	4(0.1)^a^		
Cohabitation	4(0.1)^a^	1(0.0)^a^			3(0.0)^a^	2(0.0)^a^			4(0.1)^a^	1(0.0)^a^		
**Social support**	42.85(8.85)	38.50(8.80)	4147966.000	0.000	43.56(8.59)	37.45(8.57)	3544877.500	0.000	43.05(8.77)	38.07(8.74)	3899575.500	0.000
Objective support	9.93(3.64)	8.81(3.45)	4734291.000	0.000	10.10(3.65)	8.58(3.33)	4446537.000	0.000	10.02(3.63)	8.65(3.39)	4491048.500	0.000
Subjective support	24.85(5.37)	22.45(5.45)	4246850.000	0.000	25.30(5.19)	21.78(5.35)	3631880.000	0.000	24.94(5.33)	22.25(5.44)	4047942.500	0.000
Support utility	8.06(2.08)	7.24(1.91)	4459075.000	0.000	8.17(2.07)	7.09(1.86)	4102739.000	0.000	8.09(2.08)	7.17(1.89)	4260975.000	0.000

*If the superscript letters of the two groups are the same (both a or b), the difference between the two groups was not statistically significant. If the superscript letters are different between the two groups (a and b), the difference between the two groups was statistically significant. The colored parameter indicated statistically significant*.

**Table 3:** Binary logistic regression analysis of variables.

**Variable**	**Anxiety**					**Depression**					**Sleep disorders**				
	**B**	**OR**	***p*-value**	**CI(95%)**		**B**	**OR**	***p*-value**	**CI(95%)**		**B**	**OR**	***p*-value**	**CI(95%)**	
**Gender**															
Male	−0.18	0.835	0.062	(0.687	0.938)	−0.165	0.848	0.113	(0.667	0.917)	−0.107	0.899	0.227	(0.756	1.069)
**Age (years)**			0.031					0.152					0.045		
<20	0.152	1.164	0.828	(0.317	3.087)	1.473	4.362	0.168	(0.873	21.857)	0.478	1.613	0.487	(0.419	6.212)
20–30	−0.076	0.927	0.908	(0.293	2.512)	1.545	4.690	0.135	(0.716	16.425)	0.337	1.401	0.604	(0.392	5.008)
30–40	0.104	1.109	0.874	(0.395	3.392)	1.677	5.350	0.105	(0.87	20.009)	0.530	1.698	0.414	(0.476	6.058)
40–50	0.23	1.258	0.726	(0.519	4.435)	1.517	4.559	0.142	(0.89	20.438)	0.633	1.884	0.329	(0.528	6.721)
50–60	−0.143	0.867	0.831	(0.439	3.927)	1.266	3.545	0.227	(0.773	18.437)	0.499	1.648	0.451	(0.449	6.039)
**Marital status**			0.01					0.106					0.013		
Unmarried	0.797	2.219	0.611	(0.237	2.199)	0.009	1.009	0.996	(0.147	1.485)	0.555	1.741	0.678	(0.127	23.819)
Married	1.07	2.915	0.495	(0.217	1.352)	0.175	1.192	0.917	(0.193	1.204)	0.854	2.350	0.522	(0.172	32.072)
Divorced	0.461	1.585	0.771	(0.287	1.754)	−0.456	0.634	0.789	(0.269	1.645)	0.967	2.629	0.474	(0.187	36.97)
Death of a spouse	0.628	1.873	0.729	(0.334	2.03)	−0.027	0.973	0.989	(0.34	2.065)	0.120	1.128	0.938	(0.054	23.767)
**Profession as doctor**	−0.099	0.905	0.213	(0.713	0.929)	0.078	1.081	0.370	(0.835	1.093)	−0.076	0.927	0.299	(0.802	1.07)
**First-line department**	0.239	1.27	0.000	(1.138	1.414)	0.384	1.468	0.000	(1.226	1.531)	0.406	1.501	0.000	(1.331	1.694)
**Income level per year**			0.000					0.020					0.335		
<50,000	−0.146	0.864	0.851	(0.288	3.557)	−0.742	0.476	0.322	(0.222	2.617)	0.015	1.015	0.983	(0.254	4.051)
50,000–100,000	0.098	1.103	0.899	(0.332	4.068)	−0.574	0.563	0.442	(0.238	2.786)	0.072	1.075	0.918	(0.27	4.283)
100,000–150,000	0.439	1.552	0.574	(0.429	5.359)	−0.243	0.784	0.748	(0.284	3.401)	0.300	1.350	0.674	(0.334	5.453)
150,000–200,000	0.011	1.011	0.989	(0.285	3.883)	−0.641	0.527	0.417	(0.168	2.21)	−0.049	0.952	0.947	(0.224	4.055)
200,000–300,000	−0.581	0.559	0.502	(0.114	1.85)	−0.615	0.541	0.465	(0.145	2.174)	−0.149	0.862	0.848	(0.189	3.941)
300,000–400,000	−1.075	0.341	0.305	(0.127	3.416)	0.064	1.066	0.947	(0.13	3.429)	−0.407	0.665	0.660	(0.108	4.089)
**Smoking**	−0.005	0.995	0.968	(0.863	1.328)	0.116	1.123	0.420	(0.919	1.427)	0.065	1.068	0.590	(0.842	1.354)
**Alcohol**	−0.06	0.942	0.724	(0.909	1.545)	−0.065	0.937	0.727	(1.09	1.869)	0.106	1.111	0.482	(0.828	1.493)
**Exercise**			0.000					0.000					0.011		
Never exercise	0.716	2.045	0.000	(1.59	2.66)	0.683	1.979	0.000	(1.584	2.694)	0.443	1.557	0.002	(1.175	2.065)
Irregular physical activity	0.459	1.583	0.001	(1.289	2.024)	0.350	1.419	0.022	(1.211	1.935)	0.274	1.315	0.029	(1.029	1.68)
Regular sports activities <20 min twice a week	0.21	1.234	0.2	(0.985	1.667)	0.120	1.127	0.503	(0.928	1.6)	0.118	1.125	0.422	(0.844	1.5)
Regular sports activities >20 min twice a week	0.33	1.39	0.047	(0.959	1.648)	0.168	1.183	0.358	(0.847	1.493)	0.151	1.163	0.316	(0.866	1.562)
Objective support	−0.014	0.986	0.2	(0.952	0.986)	−0.031	0.969	0.007	(0.948	0.982)	−0.039	0.962	0.000	(0.944	0.981)
Subjective support	−0.043	0.958	0.000	(0.932	0.953)	−0.057	0.945	0.000	(0.905	0.927)	−0.054	0.947	0.000	(0.935	0.96)
Support utility	−0.081	0.922	0.000	(0.865	0.917)	−0.099	0.906	0.000	(0.833	0.885)	−0.097	0.907	0.000	(0.878	0.938)

## Discussion

The psychological health of medical staff guarantees effective and persistent defense against epidemics or other chronic diseases such as cancer or dementia (Busis et al., 2017; Zhou et al., 2017). However, during the COVID-19 epidemic, physicians were confronted with increasingly prevalent burnout (The, 2019a). In addition to work-related exhaustion, Chinese medical staff became aware that there were conflicting relationships between doctors and patients, already recognized in the period 2013–2016 (Cai et al., 2019) and emphasized in 2019, which emerged from events reported in the news and from the medical society (Chen et al., 2020). This situation also received attention and solidarity from the Lancet (The, 2020). The exhaustion of medical staff was rarely considered by the public, and the more established social and political policy aspects were avoided by the media and public. However, the COVID-19 outbreak and the heroic behavior of Doctor Wenliang Li reminded the majority of Chinese society and media that medical staff represent the backbone of this battle against COVID-19. In this report, based on the surveyed result, we urge for manageable protective factors against the psychological distress of medical staff. Also the persistent concern for social respect, acceptance, and care of medical staff both in China and world wide (The, 2019b) need to be considered.

In our survey, as indicated in Figure 3, the incidence of anxiety, depression, and sleep disorders in Chinese medical staff was 35, 36, and 37%, respectively. The result was in accordance with a meta-analysis review on a large population based on 66 studies with 221,970 subjects, which showed an overall pooled prevalence of depression, anxiety, and insomnia of 31.4, 31.9, and 37.9%, respectively (Wu et al., 2021). Meanwhile, the incidences of psychological symptoms were not consistent. Take anxiety for example, it was reported as 74% (Shrestha, 2020) in a tertiary care center in Nepal, 23% in Wuhan (Huang J. Z. et al., 2020), 11.4% in Gansu (Zhu et al., 2020), and 30.4% (Liang et al., 2020) in a hospital-based survey outside Wuhan in China. The reported differences might also be explained by sampling criteria, but in any case it is important to highlight the high incidence of acute distress including depression, anxiety, and sleep disorders among medical workers during COVID-19.

Among risk factors, being female was related to a higher risk for developing psychological symptoms, which is consistent with other Chinese surveys (Li G. et al., 2020; Wang et al., 2021). It is reported that medical staff aged 21–40 years are in a more vulnerable position in terms of their mental health (Ahmed et al., 2020), and we could also see that the middle age group was more vulnerable to psychological symptoms in our survey (as shown in Figure 4 and Table 3). Working in a first-line department was also a risk factor. Understandably, medical staff standing at the front line should receive more care to protect them from mental disorders (Zhan et al., 2020). Lack of exercise was a risk factor for all the psychological symptoms (as shown in Figure 4 and Table 3), and the benefit of exercise for mental health was recognized (Mikkelsen et al., 2017). A low-income level was a risk factor for psychological symptoms in our survey. Medical staff with low income, medium-low income, and very low income levels were all susceptible to anxiety, staff with a medium-low income level were also vulnerable to depression and sleep disorders. It has been reported that income level is related to mental health both in developed countries such as Canada (Bartram, 2019) and developing countries such as Turkey (Kose, 2020).

Among the protective factors of psychological symptoms, social support was a manageable element, which we chose to discuss in detail. The high prevalence of anxiety and depression was also related to social media exposure when social support was scarce (Gao et al., 2020). Continued acknowledgment of medical staff by hospital management and the government, provision of infection control guidelines, specialized equipment, and facilities for the management of COVID-19 infection should be recognized as factors that may encourage medical staff to work during future epidemics (Cai et al., 2020). Also, the psychological symptoms brought about by the epidemic could be eased by enhancing social support (as shown in Figure 4 and Tables 1, 2), which may be adoptable in the current situation. The Chinese national culture has its intrinsic characteristics of emphasizing the social value of the individual and a consciousness-like “mianzi.” “Mianzi” can be translated as “face,” but connotes more dignity and respect rather than the physical organ of the face. This is also a double-edged sword, due to the emphasis of external acceptance, people put the group requirements of society first and inner personal requirements second. This custom and practice could lead to quick action during emergencies such as an epidemic but also a high incidence of psychological symptoms of susceptible individuals. Social support means a social structure that does not judge or blame but listens and comforts (shown in the translated version of SSS in the Supplementary Material). The concept and realization of social support could be an effective method to improve nursing quality. Compared with money as a typical objective support element, which was the only protective element for sleep disorders, subjective support and support utility were more essential and stable protective factors for medical staff for anxiety, depression, and sleep disorders (as shown in Figure 4 and Table 3). A paralleled study showed that initiated and sustained person-centered communication as subjective support could ease both the psychological distress of the medical staff and infected older adults despite multiple challenges brought by the pandemic (Li J. et al., 2020). Thus, we also believe that social support will be helpful for the public. In addition to social support, exercise was also an adaptive way to enhance immunity for the fight against the epidemic. As indicated in our survey, getting more than 20 min of exercise per day should be encouraged and implemented by the medical staff dealing with the epidemic to protect them from anxiety and depression (as shown in Figure 4 and Table 3). Exercise should also be adopted into daily life to maintain mental health (Deslandes et al., 2009).

## Conclusion

The results indicated that the medical staff had a high incidence of psychological symptoms, which were more prominent during the COVID-19 outbreak. Comparatively, being female, a nurse, working in a first-line department, never exercising, and having a low income were risk factors for psychological symptoms. Social support including objective support, subjective support, support utility, and regular exercise over 3 times per week were found to be elements that could protect the medical staff against psychological symptoms. In conclusion, the susceptibility of psychological symptoms of medical staff should raise the concern of both policymakers and the public in the long-term, and the aggravation of mental health problems of medical staff should be eased by providing adequate social support during and after the COVID-19 pandemic.

## Data Availability Statement

The original contributions presented in the study are included in the article/Supplementary Material, further inquiries can be directed to the corresponding authors.

## Ethics Statement

The studies involving human participants were reviewed and approved by Ethic committee of Hainan general hospital. The patients/participants provided their electrical informed consent to participate in this study.

## Author Contributions

HLL: acquisition of data, analysis and interpretation, and critical revision of the manuscript for important intellectual content. HQY: study concept and design, analysis and interpretation of data analysis, and interpretation of data. YDX: analysis and interpretation, and critical revision of the manuscript for important intellectual content. ZZ, JiaL, JieL, and XWW: acquisition of data. ZXZ: study concept, acquisition of data, analysis and interpretation, critical revision of the manuscript for important intellectual content, and study supervision. YL: study concept and design, analysis and interpretation of data, analysis and interpretation of data analysis, critical revision of the manuscript, and supervision of the study. All authors listed have contributed sufficiently to the project to be included as authors, and all those who are qualified to be authors are listed in the author byline.

## Conflict of Interest

The authors declare that the research was conducted in the absence of any commercial or financial relationships that could be construed as a potential conflict of interest.
