# Supplementary material for: Protective and Risk Factors for Medical and Nursing Staff Suffering From Psychological Symptoms During COVID-19
Source: Front Psychol. 2021 Apr 16;12:603553. doi: 10.3389/fpsyg.2021.603553 (PMC8086510; doi:10.3389/fpsyg.2021.603553)
Supplement: Supplementary file 1 [file Data_Sheet_1.docx]

Social Support Scale

1. How many friends do you have that are close and can get support and help? [Multiple choice questions]*

| ○None |
| --- |
| ○1-2 |
| ○3-5 |
| ○6 or above 6 |

2. In the past year you: [Single choice] *

| ○Stay away from family and live alone |
| --- |
| ○The residence often changes, living with strangers most of the time |
| ○Live with classmates, colleagues or friends |
| ○Live with family |

3. You and your neighbor: [Single choice] *

| ○Never cared about each other, just nodded |
| --- |
| ○May be slightly concerned about difficulties |
| ○Some neighbors care about you |
| ○Most neighbors care about you |

4. You and your colleagues: [Single choice] *

| ○Never cared about each other, just nodded |
| --- |
| ○May be slightly concerned about difficulties |
| ○Some colleagues care about you |
| ○Most colleagues care about you |

5. Support and care received from family members: [matrix scale question] *

|  | None | Rarely | General | Full support |
| --- | --- | --- | --- | --- |
| Couple (lover) | ○ | ○ | ○ | ○ |
| Parents | ○ | ○ | ○ | ○ |
| Children | ○ | ○ | ○ | ○ |
| Brothers and Sisters | ○ | ○ | ○ | ○ |
| Other family members | ○ | ○ | ○ | ○ |

6. In the past, when you encountered an emergency situation, the sources of financial support and help to solve practical problems were: [multiple choice] *

| □No source |
| --- |
| □Spouse |
| □Other family members |
| □Relatives |
| □Friends |
| □Colleagues |
| □Work unit |
| □Official, or semi-official organizations such as party unions |
| □Religious organizations |
| □Others _________________* |

7. In the past, when you encountered an emergency situation, the sources of comfort and concern you have received are: [Multiple choice] *

| □No source |  |
| --- | --- |
| □Spouse |  |
| □Other family members |  |
| □Relatives |  |
| □Friends |  |
| □Colleagues |  |
| □Work unit |  |
| □Official, or semi-official organizations such as party unions |  |
| □Religious organizations |  |
| □Others _________________* |  |

8. In the past, when you encountered an emergency situation, the sources of comfort and concern you have received are: [Single choice] *

| ○Never complain to anyone |
| --- |
| ○Only complain to 1-2 people who are very close |
| ○If a friend asks you, you will say it |
| ○Proactively report your worries for support and understanding |

9. How to ask for help when you are in trouble: [Single choice] *

| ○Rely on oneself alone, not accepting help from others |
| --- |
| ○Rarely ask for help |
| ○Sometimes ask for help |
| ○Always ask for help from family, relatives, and organizations when in trouble |

10. For groups (such as party organizations, religious organizations, trade unions, student unions, etc.) to organize activities, you: [Multiple questions] *

| ○Never participate |
| --- |
| ○Participate occasionally |
| ○Attend often |
| ○Active in participating |

Note:

1. Objective support dimension: 2, 6, 7 (according to the item 27, 31, 32 in our survey)
2. Subjective support dimension: 1, 3, 4, 5 (according to the item 26, 28, 29, 30 in our survey)
3. Support utility dimension: 8, 9, 10 (according to the item 33, 34, 35 in our survey)
