# Supplementary material for: Protective and Risk Factors for Medical and Nursing Staff Suffering From Psychological Symptoms During COVID-19
Source: Front Psychol. 2021 Apr 16;12:603553. doi: 10.3389/fpsyg.2021.603553 (PMC8086510; doi:10.3389/fpsyg.2021.603553)
Supplement: Supplementary file 2 [file Data_Sheet_2.docx]

**新型冠状病毒疫情下医务人员的睡眠和心理状况的评估**

      本问卷（共36题）旨在了解新型冠状病毒疫情对医务人员的睡眠和心理相关状况的影响，以及目前的社会支持情况，并根据评估结果为您提供个性化的干预措施。
     本问卷采取匿名方式，保证信息的绝对保密。您所提供的答案不存在对错之分，只要真实反映您的实际情况即可。再次感谢您的参与！

1. 您的性别： [单选题] *

| ○男 | ○女 |  |  |  |  |  |  |
| --- | --- | --- | --- | --- | --- | --- | --- |

2. 您的年龄段（周岁）： [单选题] *

麻烦填上您的实龄（数字）

| ○≤20 _________________ * | ○21~30 _________________ * |
| --- | --- |
| ○31~40 _________________ * | ○41~50 _________________ * |
| ○51~60 _________________ * | ○＞60 _________________ * |

3. 婚姻状况 [单选题] *

| ○未婚 | ○已婚 | ○离异 | ○丧偶 | ○同居 |
| --- | --- | --- | --- | --- |

4. 文化程度 [单选题] *

| ○高中以下 | ○中专 | ○大专 | ○本科 | ○硕士 | ○博士、博士后 |
| --- | --- | --- | --- | --- | --- |

5. 职业： [单选题] *

| ○医师 | ○护士 | ○技师 | ○药师 | ○其他 |
| --- | --- | --- | --- | --- |

6. 所在科室： [单选题] *

| ○一线(感染、发热门诊、急诊、呼吸、重症监护、全科) |
| --- |
| ○二线(其他科室) |

7. 收入状况： [单选题] *

| ○＜5万 | ○5-10万 | ○11-15万 | ○16-20万 | ○21-30万 | ○31-40万 | ○＞40万 |
| --- | --- | --- | --- | --- | --- | --- |

8. 是否吸烟： [单选题] *

| ○是 |
| --- |
| ○否 |

9. 是否饮酒(白酒一次达一两或啤酒200ml，每周两次以上，持续6个月)： [单选题] *

| ○是 |
| --- |
| ○否 |

10. 是否有慢性疾病(高血压、糖尿病、心脑血管疾病、呼吸系统疾病等)： [单选题] *

| ○是 |
| --- |
| ○否 |

11. 甲状腺功能： [单选题] *

| ○正常 |
| --- |
| ○异常 |

12. 是否有精神疾病： [单选题] *

| ○无 | ○抑郁症 | ○焦虑症 | ○强迫症 | ○精神分裂症 | ○双相情感障碍 | ○其他 _________________ * |
| --- | --- | --- | --- | --- | --- | --- |

13. 平时体育运动情况 [单选题] *

| ○从不运动 |
| --- |
| ○无规律体育运动 |
| ○每周2次 20分钟以上的运动 |
| ○每周3-4次20分钟以上的运动 |
| ○每周＞5次 20分钟以上的运动 |

14. 在**最近两周**，您有多少时间受到以下问题困扰？[矩阵量表题] *

|  | 完全不会 | 几天 | 一半以上的日子 | 几乎每天 |
| --- | --- | --- | --- | --- |
| 感觉紧张，焦虑或急切 | ○ | ○ | ○ | ○ |
| 不能够停止或控制担忧 | ○ | ○ | ○ | ○ |
| 对各种各样的事情担忧过多 | ○ | ○ | ○ | ○ |
| 很难放松下来 | ○ | ○ | ○ | ○ |
| 由于不安而无法静坐 | ○ | ○ | ○ | ○ |
| 变得容易烦恼或急躁 | ○ | ○ | ○ | ○ |
| 感到似乎将有可怕的事情发生而害怕 | ○ | ○ | ○ | ○ |

15. 在**最近两周**，您有多少时间受到以下问题的困扰？[矩阵量表题] *

|  | 没有 | 有几天 | 一半以上时间 | 几乎每天 |
| --- | --- | --- | --- | --- |
| 做事时提不起劲或没有兴趣 | ○ | ○ | ○ | ○ |
| 感到心情低落、沮丧或绝望 | ○ | ○ | ○ | ○ |
| 入睡困难、睡不安稳或睡眠过多 | ○ | ○ | ○ | ○ |
| 感觉疲倦或没有活力 | ○ | ○ | ○ | ○ |
| 食欲不振或吃太多 | ○ | ○ | ○ | ○ |
| 觉得自己很糟，或觉得自己很失败，或让自己或家人失望 | ○ | ○ | ○ | ○ |
| 对事物专注有困难，例如阅读报纸或看电视时不能集中注意力 | ○ | ○ | ○ | ○ |
| 动作或说话速度缓慢到别人已经觉察？（或正好相反，烦躁或坐立不安、动来动去的情况更胜于平常） | ○ | ○ | ○ | ○ |
| 有不如死掉或用某种方式伤害自己的念头 | ○ | ○ | ○ | ○ |

16. 描述您**当前（或最近一周）**失眠问题的严重程度[矩阵量表题] *

|  | 无 | 轻度 | 中度 | 重度 | 极重度 |
| --- | --- | --- | --- | --- | --- |
| 入睡困难 | ○ | ○ | ○ | ○ | ○ |
| 维持睡眠困难 | ○ | ○ | ○ | ○ | ○ |
| 早醒 | ○ | ○ | ○ | ○ | ○ |

17. 对您**当前**睡眠的满意程度 [单选题] *

| 很满意 | ○0 | ○1 | ○2 | ○3 | ○4 | 很不满意 |
| --- | --- | --- | --- | --- | --- | --- |

18. 您认为您**当前**的睡眠问题在多大程度上干扰了您的日间功能（如日间疲劳、处理工作和日常事务的能力、注意力、记忆力、情绪等） [单选题] *

| 没有干扰 | ○0 | ○1 | ○2 | ○3 | ○4 | 很多干扰 |
| --- | --- | --- | --- | --- | --- | --- |

19. **当前（或最近一周）**[矩阵量表题] *

|  | 没有 | 一点 | 有些 | 较多 | 很多 |
| --- | --- | --- | --- | --- | --- |
| 与其他人相比，您的失眠问题对您的生活质量有多大程度的影响或损害 | ○ | ○ | ○ | ○ | ○ |
| 您对自己当前睡眠问题有多大程度的焦虑和烦扰 | ○ | ○ | ○ | ○ | ○ |

20. 在**一个月以前**（***2020年1月20日以前***），您有多少时间受到以下问题困扰？[矩阵量表题] *

|  | 完全不会 | 几天 | 一半以上的日子 | 几乎每天 |
| --- | --- | --- | --- | --- |
| 感觉紧张，焦虑或急切 | ○ | ○ | ○ | ○ |
| 不能够停止或控制担忧 | ○ | ○ | ○ | ○ |
| 对各种各样的事情担忧过多 | ○ | ○ | ○ | ○ |
| 很难放松下来 | ○ | ○ | ○ | ○ |
| 由于不安而无法静坐 | ○ | ○ | ○ | ○ |
| 变得容易烦恼或急躁 | ○ | ○ | ○ | ○ |
| 感到似乎将有可怕的事情发生而害怕 | ○ | ○ | ○ | ○ |

21. 在**一个月以前（2020年1月20日以前）**，您有多少时间受到以下问题的困扰？[矩阵量表题] *

|  | 没有 | 有几天 | 一半以上时间 | 几乎每天 |
| --- | --- | --- | --- | --- |
| 做事时提不起劲或没有兴趣 | ○ | ○ | ○ | ○ |
| 感到心情低落、沮丧或绝望 | ○ | ○ | ○ | ○ |
| 入睡困难、睡不安稳或睡眠过多 | ○ | ○ | ○ | ○ |
| 感觉疲倦或没有活力 | ○ | ○ | ○ | ○ |
| 食欲不振或吃太多 | ○ | ○ | ○ | ○ |
| 觉得自己很糟，或觉得自己很失败，或让自己或家人失望 | ○ | ○ | ○ | ○ |
| 对事物专注有困难，例如阅读报纸或看电视时不能集中注意力 | ○ | ○ | ○ | ○ |
| 动作或说话速度缓慢到别人已经觉察？（或正好相反，烦躁或坐立不安、动来动去的情况更胜于平常） | ○ | ○ | ○ | ○ |
| 有不如死掉或用某种方式伤害自己的念头 | ○ | ○ | ○ | ○ |

22. 描述您**一个月前（2020年1月20日前）**失眠问题的严重程度[矩阵量表题] *

|  | 无 | 轻度 | 中度 | 重度 | 极重度 |
| --- | --- | --- | --- | --- | --- |
| 入睡困难 | ○ | ○ | ○ | ○ | ○ |
| 维持睡眠困难 | ○ | ○ | ○ | ○ | ○ |
| 早醒 | ○ | ○ | ○ | ○ | ○ |

23. 对您**一个月前（2020年1月20日前）**睡眠的满意程度 [单选题] *

| 很满意 | ○0 | ○1 | ○2 | ○3 | ○4 | 很不满意 |
| --- | --- | --- | --- | --- | --- | --- |

24. 您认为您**一个月前（2020年1月20日前）**的睡眠问题在多大程度上干扰了您的日间功能（如日间疲劳、处理工作和日常事务的能力、注意力、记忆力、情绪等） [单选题] *

| 没有干扰 | ○0 | ○1 | ○2 | ○3 | ○4 | 很多干扰 |
| --- | --- | --- | --- | --- | --- | --- |

25. **一个月前（2020年1月20日前）**[矩阵量表题] *

|  | 没有 | 一点 | 有些 | 较多 | 很多 |
| --- | --- | --- | --- | --- | --- |
| 与其他人相比，您的失眠问题对您的生活质量有多大程度的影响或损害 | ○ | ○ | ○ | ○ | ○ |
| 您对自己一个月前的睡眠问题有多大程度的焦虑和烦扰 | ○ | ○ | ○ | ○ | ○ |

26. 您有多少关系密切，可以得到支持和帮助的朋友？ [单选题] *

| ○一个也没有 |
| --- |
| ○1-2个 |
| ○3-5个 |
| ○6个或6个以上 |

27. 近一年来您： [单选题] *

| ○远离家人，且独居一室 |
| --- |
| ○住处经常变动，多数时间和陌生人住在一起 |
| ○和同学、同事或朋友住在一起 |
| ○和家人住在一起 |

28. 您与邻居： [单选题] *

| ○相互之间从不关心，只是点头之交 |
| --- |
| ○遇到困难可能稍微关心 |
| ○有些邻居都很关心您 |
| ○大多数邻居都很关心您 |

29. 您与同事： [单选题] *

| ○相互之间从不关心，只是点头之交 |
| --- |
| ○遇到困难可能稍微关心 |
| ○有些同事很关心您 |
| ○大多数同事都很关心您 |

30. 从家庭成员得到的支持和照顾：[矩阵量表题] *

|  | 无 | 极少 | 一般 | 全力支持 |
| --- | --- | --- | --- | --- |
| 夫妻（恋人） | ○ | ○ | ○ | ○ |
| 父母 | ○ | ○ | ○ | ○ |
| 儿女 | ○ | ○ | ○ | ○ |
| 兄弟姐妹 | ○ | ○ | ○ | ○ |
| 其他成员（如嫂子） | ○ | ○ | ○ | ○ |

31. 过去，在您遇到急难情况时，曾经得到的经济支持和解决实际问题的帮助的来源有： [多选题] *

| □无任何来源 |
| --- |
| □配偶 |
| □其他家人 |
| □亲戚 |
| □朋友 |
| □同事 |
| □工作单位 |
| □党团工会等官方或半官方组织 |
| □宗教、社会团体等非宫方组织 |
| □其它 _________________* |

32. 过去，在您遇到急难情况时，曾经得到的安慰和关心的来源有： [多选题] *

| □无任何来源 |
| --- |
| □配偶 |
| □其他家人 |
| □亲戚 |
| □朋友 |
| □同事 |
| □工作单位 |
| □党团工会等官方或半官方组织 |
| □宗教、社会团体等非宫方组织 |
| □其它 _________________* |

33. 您遇到烦恼时的倾诉方式： [单选题] *

| ○从不向任何人诉述 |
| --- |
| ○只向关系极为密切的1-2个人诉述 |
| ○如果朋友主动询问您会说出来 |
| ○主动诉述自己的烦恼，以获得支持和理解 |

34. 您遇到烦恼时的求助方式： [单选题] *

| ○只靠自己，不接受别人帮助。 |
| --- |
| ○很少请求别人帮助。 |
| ○有时请求别人帮助。 |
| ○有困难时经常向家人、亲友、组织求援。 |

35. 对于团体(如党团组织、宗教组织、工会、学生会等)组织活动，您： [单选题] *

| ○从不参加 |
| --- |
| ○偶尔参加 |
| ○经常参加 |
| ○主动参加并积极活动 |

36. 您的文化程度是： [单选题] *

| ○高中以下 |
| --- |
| ○中专 |
| ○大专 |
| ○本科 |
| ○硕士 |
| ○博士、博士后 |
