# Supplementary material for: Protective and Risk Factors for Medical and Nursing Staff Suffering From Psychological Symptoms During COVID-19
Source: Front Psychol. 2021 Apr 16;12:603553. doi: 10.3389/fpsyg.2021.603553 (PMC8086510; doi:10.3389/fpsyg.2021.603553)
Supplement: Supplementary file 3 [file Data_Sheet_3.docx]

**Survey of Sleeping and Psychological Status of Medical Staff in COVID-19**

      This questionnaire (36 questions) aims to understand the impact of the new coronavirus epidemic on the sleep and psychological-related conditions of medical staff, as well as the current social support situation, and to provide you with personalized interventions based on the results of the assessment.

      This questionnaire is taken anonymously to ensure the absolute confidentiality of the information. There is no right or wrong answer provided by you, as long as it truly reflects your actual situation. Thanks again for your participation!

1. Your gender: [Multiple choice]： [Single choice] *

| ○Male | ○Female |  |  |  |  |  |  |
| --- | --- | --- | --- | --- | --- | --- | --- |

2. Your age（full year of life）： [Single choice] *

| ○≤20 _________________ * | ○21~30 _________________ * |
| --- | --- |
| ○31~40 _________________ * | ○41~50 _________________ * |
| ○51~60 _________________ * | ○＞60 _________________ * |

3. Marriage status [Single choice] *

| ○Umarried | ○Married | ○Divorce | ○Widowed | ○Cohabitation |
| --- | --- | --- | --- | --- |

4. Educational level [Single choice] *

| ○Secondary school | ○Technical secondary school | ○College | ○Undergraduate | ○Post graduate | ○Phd and above |
| --- | --- | --- | --- | --- | --- |

5. Professional： [Single choice] *

| ○Clinical doctors | ○Nurses | ○Technician | ○Phamacist | ○Others |
| --- | --- | --- | --- | --- |

6. Department： [Single choice] *

| ○First line (infection, fever, emergency, respiratory, intensive care, general practice) |
| --- |
| ○Second line (other departments) |

7. Income level(RMB)： [Single choice] *

| ○＜50,000 | ○50,000-100,000 | ○100,000-150,000 | ○150,000-200,000 | ○200,000-300,000 | ○300,000-400,000 | ○＞400,000 |
| --- | --- | --- | --- | --- | --- | --- |

8. Smoking status： [Single choice] *

| ○yes |
| --- |
| ○no |

9. Alcohol(Liquor up to one or two at a time or 200ml of beer, more than twice a week for 6 months)： [Single choice] *

| ○yes |
| --- |
| ○no |

10. Are there any chronic diseases (hypertension, diabetes, cardiovascular and cerebrovascular diseases, respiratory diseases, etc.)： [Single choice] *

| ○yes |
| --- |
| ○no |

11. Thyroid function： [Single choice] *

| ○Normal |
| --- |
| ○Abnormal |

12. Past history of mental disease： [Single choice] *

| ○No | ○Depression | ○Anxiety | ○OCD | ○Schizophrenia | ○Bipolar Disorder | ○Others _________________ * |
| --- | --- | --- | --- | --- | --- | --- |

13. Public sports situation [Single choice] *

| ○Never |
| --- |
| ○No regular sports activities |
| ○Exercise twice a week for 20 minutes or more |
| ○Exercise more than 20 minutes 3-4 times a week |
| ○> Exercise 5 times a week for more than 20 minutes |

14. How much time have you been plagued by the following questions **in the last two weeks**? [Matrix scale questions] *

|  | Not at all | Several days | More than half the days | Almost every day |
| --- | --- | --- | --- | --- |
| Feeling nervous, anxious or eager | ○ | ○ | ○ | ○ |
| Can't stop or control worry | ○ | ○ | ○ | ○ |
| Too much worry about various things | ○ | ○ | ○ | ○ |
| Difficult to relax | ○ | ○ | ○ | ○ |
| Unable to sit still because of restlessness | ○ | ○ | ○ | ○ |
| Become easily upset or irritable | ○ | ○ | ○ | ○ |
| Feeling afraid that something terrible will happen | ○ | ○ | ○ | ○ |

15. How much time have you been plagued by the following questions **in the last two weeks**? [Matrix scale questions] *

|  | Not at all | Several days | More than half the days | Almost every day |
| --- | --- | --- | --- | --- |
| Unable to show interest or interest in doing things? | ○ | ○ | ○ | ○ |
| Feeling down, depressed or hopeless | ○ | ○ | ○ | ○ |
| Difficulty falling asleep, restlessness, or excessive sleep | ○ | ○ | ○ | ○ |
| Feeling tired or inactive | ○ | ○ | ○ | ○ |
| Loss of appetite or eating too much | ○ | ○ | ○ | ○ |
| Feeling bad, or failing, or disappointing yourself or your family | ○ | ○ | ○ | ○ |
| Difficulty focusing on things, such as not being able to pay attention while reading a newspaper or watching TV | ○ | ○ | ○ | ○ |
| Movement or speaking speed is so slow that others have already noticed it? (Or vice versa, irritability, restlessness, and movement are better than usual) | ○ | ○ | ○ | ○ |
| Thoughts of dying or hurting yourself in some way | ○ | ○ | ○ | ○ |

16. Describe the severity of your **current (or recent week)** insomnia problem [Matrix scale questions] *

|  | No | Mild | Moderate | Severe | Extremely severe |
| --- | --- | --- | --- | --- | --- |
| difficulty falling asleep | ○ | ○ | ○ | ○ | ○ |
| Difficulty maintaining sleep | ○ | ○ | ○ | ○ | ○ |
| Wake up early | ○ | ○ | ○ | ○ | ○ |

17. Satisfaction with your **current** sleep [Single choice] *

| Very satisfied | ○0 | ○1 | ○2 | ○3 | ○4 | Very dissatisfied |
| --- | --- | --- | --- | --- | --- | --- |

18. To what extent do you think your **current** sleep problems are interfering with your daytime functions (such as daytime fatigue, ability to handle work and daily affairs, attention, memory, mood, etc.) [Single choice] *

| No interference | ○0 | ○1 | ○2 | ○3 | ○4 | a lot of interference |
| --- | --- | --- | --- | --- | --- | --- |

19. **Current (or last week)** [Matrix scale questions] *

|  | No | A little | Some | Much | Too much |
| --- | --- | --- | --- | --- | --- |
| How much your insomnia affects or impairs your quality of life compared to others | ○ | ○ | ○ | ○ | ○ |
| How much anxiety and annoyance you have about your current sleep problems | ○ | ○ | ○ | ○ | ○ |

20. **A month ago (before January 20, 2020)**, how much time did you suffer from the following questions? [Matrix scale question] *

|  | Not at all | Several days | More than half the days | Almost every day |
| --- | --- | --- | --- | --- |
| Feeling nervous, anxious or eager | ○ | ○ | ○ | ○ |
| Can't stop or control worry | ○ | ○ | ○ | ○ |
| Too much worry about various things | ○ | ○ | ○ | ○ |
| Difficult to relax | ○ | ○ | ○ | ○ |
| Unable to sit still because of restlessness | ○ | ○ | ○ | ○ |
| Become easily upset or irritable | ○ | ○ | ○ | ○ |
| Feeling afraid that something terrible will happen | ○ | ○ | ○ | ○ |

21. **A month ago (before January 20, 2020)**, how much time did you suffer from the following questions? [Matrix scale question] *

|  | Not at all | Several days | More than half the days | Almost every day |
| --- | --- | --- | --- | --- |
| Unable to show interest or interest in doing things? | ○ | ○ | ○ | ○ |
| Feeling down, depressed or hopeless | ○ | ○ | ○ | ○ |
| Difficulty falling asleep, restlessness, or excessive sleep | ○ | ○ | ○ | ○ |
| Feeling tired or inactive | ○ | ○ | ○ | ○ |
| Loss of appetite or eating too much | ○ | ○ | ○ | ○ |
| Feeling bad, or failing, or disappointing yourself or your family | ○ | ○ | ○ | ○ |
| Difficulty focusing on things, such as not being able to pay attention while reading a newspaper or watching TV | ○ | ○ | ○ | ○ |
| Movement or speaking speed is so slow that others have already noticed it? (Or vice versa, irritability, restlessness, and movement are better than usual) | ○ | ○ | ○ | ○ |
| Thoughts of dying or hurting yourself in some way | ○ | ○ | ○ | ○ |

22. Describe the severity of your insomnia problem **one month ago (before January 20, 2020)** [matrix scale question] *

|  | No | Mild | Moderate | Severe | Extremely severe |
| --- | --- | --- | --- | --- | --- |
| difficulty falling asleep | ○ | ○ | ○ | ○ | ○ |
| Difficulty maintaining sleep | ○ | ○ | ○ | ○ | ○ |
| Wake up early | ○ | ○ | ○ | ○ | ○ |

23. Satisfaction with your sleep **a month ago (before January 20, 2020)** [Single-choice question] *

| Very satisfied | ○0 | ○1 | ○2 | ○3 | ○4 | Very dissatisfied |
| --- | --- | --- | --- | --- | --- | --- |

24. To what extent do you think that your sleep problem **a month ago (before January 20, 2020)** interfered with your daytime functions (such as daytime fatigue, ability to handle work and daily affairs, attention, memory, emotions, etc.) ) [Multiple choice questions]*

| No interfere | ○0 | ○1 | ○2 | ○3 | ○4 | Much interfere |
| --- | --- | --- | --- | --- | --- | --- |

25. **One month ago (before January 20, 2020)** [matrix scale question]*

|  | No | A little | Some | Much | Too much |
| --- | --- | --- | --- | --- | --- |
| How much your insomnia affects or impairs your quality of life compared to others | ○ | ○ | ○ | ○ | ○ |
| How much anxiety and annoyance you have about your current sleep problems | ○ | ○ | ○ | ○ | ○ |

26. How many friends do you have that are close and can get support and help? [Multiple choice questions]*

| ○None |
| --- |
| ○1-2 |
| ○3-5 |
| ○6 or above 6 |

27. In the past year you: [Single choice] *

| ○Stay away from family and live alone |
| --- |
| ○The residence often changes, living with strangers most of the time |
| ○Live with classmates, colleagues or friends |
| ○Live with family |

28. You and your neighbor: [Single choice] *

| ○Never cared about each other, just nodded |
| --- |
| ○May be slightly concerned about difficulties |
| ○Some neighbors care about you |
| ○Most neighbors care about you |

29. You and your colleagues: [Single choice] *

| ○Never cared about each other, just nodded |
| --- |
| ○May be slightly concerned about difficulties |
| ○Some colleagues care about you |
| ○Most colleagues care about you |

30. Support and care received from family members: [matrix scale question] *

|  | None | Rarely | General | Full support |
| --- | --- | --- | --- | --- |
| Couple (lover) | ○ | ○ | ○ | ○ |
| Parents | ○ | ○ | ○ | ○ |
| Children | ○ | ○ | ○ | ○ |
| Brothers and Sisters | ○ | ○ | ○ | ○ |
| Other family members | ○ | ○ | ○ | ○ |

31. In the past, when you encountered an emergency situation, the sources of financial support and help to solve practical problems were: [multiple choice] *

| □No source |
| --- |
| □Spouse |
| □Other family members |
| □Relatives |
| □Friends |
| □Colleagues |
| □Work unit |
| □Official, or semi-official organizations such as party unions |
| □Religious organizations |
| □Others _________________* |

32. In the past, when you encountered an emergency situation, the sources of comfort and concern you have received are: [Multiple choice] *

| □No source |  |
| --- | --- |
| □Spouse |  |
| □Other family members |  |
| □Relatives |  |
| □Friends |  |
| □Colleagues |  |
| □Work unit |  |
| □Official, or semi-official organizations such as party unions |  |
| □Religious organizations |  |
| □Others _________________* |  |

33. In the past, when you encountered an emergency situation, the sources of comfort and concern you have received are: [Single choice] *

| ○Never complain to anyone |
| --- |
| ○Only complain to 1-2 people who are very close |
| ○If a friend asks you, you will say it |
| ○Proactively report your worries for support and understanding |

34. How to ask for help when you are in trouble: [Single choice] *

| ○Rely on oneself alone, not accepting help from others |
| --- |
| ○Rarely ask for help |
| ○Sometimes ask for help |
| ○Always ask for help from family, relatives, and organizations when in trouble |

35. For groups (such as party organizations, religious organizations, trade unions, student unions, etc.) to organize activities, you: [Multiple questions] *

| ○Never participate |
| --- |
| ○Participate occasionally |
| ○Attend often |
| ○Active in participating |

36.. Educational level [Single choice] *

| ○Secondary school | ○Technical secondary school | ○College | ○Undergraduate | ○Post graduate | ○Phd and above |
| --- | --- | --- | --- | --- | --- |
